# Replication fork instability and the consequences of fork collisions from rereplication

**DOI:** 10.1101/gad.288142.116

**Published:** 2016-10-15

**Authors:** Jessica L. Alexander, Terry L. Orr-Weaver

**Affiliations:** Whitehead Institute, Department of Biology, Massachusetts Institute of Technology, Cambridge, Massachusetts 02142, USA

**Keywords:** gene amplification, double-strand break repair, nonhomologous end-joining, homologous recombination, break-induced replication

## Abstract

Replication forks encounter obstacles that must be repaired or bypassed to complete chromosome duplication before cell division. Proteomic analysis of replication forks suggests that the checkpoint and repair machinery travels with unperturbed forks, implying that they are poised to respond to stalling and collapse. However, impaired fork progression still generates aberrations, including repeat copy number instability and chromosome rearrangements. Deregulated origin firing also causes fork instability if a newer fork collides with an older one, generating double-strand breaks (DSBs) and partially rereplicated DNA. Current evidence suggests that multiple mechanisms are used to repair rereplication damage, yet these can have deleterious consequences for genome integrity.

## Abstract (precis)

In this review, Alexander and Orr-Weaver discuss replication fork instability, the mechanisms used to repair rereplication damage, and their consequences for genome integrity.

Complete and accurate duplication of DNA at each S phase is required to maintain genome integrity in dividing cells. This is accomplished by exquisite control of the DNA replication program at the level of both origin firing and replication fork progression. Genome stability requires that fork elongation is complete across every chromosome. However, not all genomic positions are replicated equally. DNA damage as well as intrinsic sequence and structural properties of the chromosome can slow or prevent passage of the replication fork. Failure to alleviate these blockades can lead to incomplete genome duplication, resulting in chromosome breakage, fusions, and rearrangements.

Many of the components that constitute the replication fork are well defined, and recent proteomics analyses have cataloged fork components during normal elongation as well as under stress conditions. Increasing evidence suggests that replication forks recruit repair components even during unstressed replication, revealing that this system is well poised to respond to fork impediments. Despite this, fork collapse and the resulting DNA damage are observed under a variety of conditions that block fork elongation. Although significant advances have been made, the mechanisms that maintain fork stability and repair damaged forks continue to be explored.

Regulated origin firing also is essential for genome integrity, and refiring of a single origin within the same S phase generates double-strand breaks (DSBs) and activates the DNA damage checkpoint. Rereplication forks are slow moving, and even multiple refired origins are not able to replicate the chromosome fully (Nguyen et al. 2001). Recent evidence reveals that DNA damage generated during rereplication is the result of instability at the replication forks, consistent with their slow progression. Continued elongation is dependent on the DNA damage checkpoint and DSB repair components. These results support the fork collision model of DSB generation during rereplication, by which adjacent forks experience head-to-tail collisions and subsequent fork collapse (Davidson et al. 2006). How these rereplication-induced DSBs are repaired is still under investigation, and current evidence varies between experimental systems. There are increasing indications that the actual repair events can exacerbate the damage depending on the location of rereplication and the mechanism of repair. These results suggest that not only fork instability but also repair of collapsed forks contribute to genome instability.

## Assembly and structure of the eukaryotic replication fork

In G1 of the cell cycle, origins of replication are bound by the prereplication complex (pre-RC). This complex includes the origin recognition complex (ORC; which directly binds to the DNA) as well as Cdc6, Cdt1, and the Mcm2–7 complex (Tanaka and Araki 2013). Cdt1 recruits Mcm2–7 to ORC and Cdc6-bound origins, and in vitro studies show that both Cdc6 and Cdt1 quickly disassociate once Mcm2–7 is stably loaded (Ticau et al. 2015). The Mcm2–7 complex is sequentially loaded as a head-to-head double hexamer onto the dsDNA origin to facilitate bidirectional fork movement (Tanaka and Araki 2013; Ticau et al. 2015). Once assembled, the origin is said to be licensed.

DDK and CDK phosphorylation events lead to the recruitment of Cdc45 and the GINS complex (Sld5, Pif1, Pif2, and Pif3), which, together with Mcm2–7, comprise the CMG (Cdc45, Mcm2–7 complex, and GINS complex) helicase that unwinds the dsDNA for replication (Tanaka and Araki 2013). Assembly of the helicase also is dependent on the regulatory components Sld2, Sld3, Sld7, and Dbp11 in budding yeast and TopBP1/Mus101, RecQL4/RecQ4, and Treslin/Ticrr in higher eukaryotes; together with the CMG and DNA polymerase ε (Pol ε), these components comprise the preinitiation complex (pre-IC) (Tanaka and Araki 2013).

**Figure 1.**
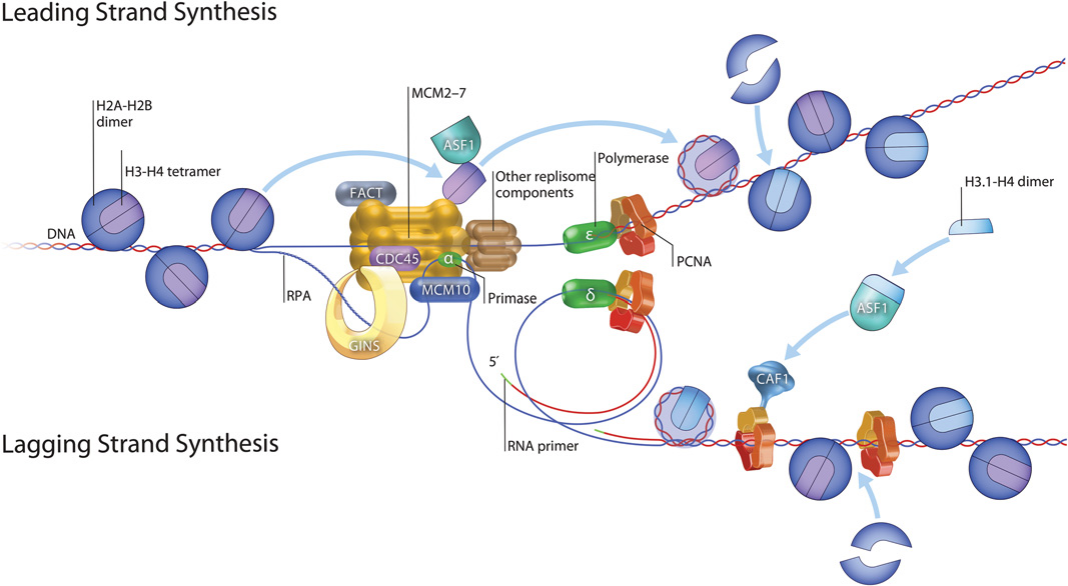
Summary of the eukaryotic DNA replication fork. Cdc45, the Mcm2–7 complex, and the GINS complex comprise the CMG helicase that unwinds the dsDNA. Leading strand synthesis is shown at the *top* and is accomplished by Pol ε. Lagging strand synthesis is depicted *below*. Pol α primase synthesizes 8- to 15-nucleotide-long RNA primers along the lagging strand. Synthesis of the lagging strand is performed by Pol δ. PCNA binds to Pol δ and Pol ε to enhance processivity. The nucleosome remodelers FACT and ASF1 bind to Mcm2–7 to coordinate removal of nucleosomes with the oncoming replication fork. ASF1 also cooperates with CAF-1 to deposit new H3–H4 tetramers behind the elongating fork. (Illustration by Steven Lee, http://www.graphiko.com. Adapted by permission from Macmillan Publishers Ltd: *Nature Reviews Molecular Cell Biology* [Alabert and Groth 2012] © 2012.)

The elongation phase of DNA replication consists of replication fork progression and DNA synthesis at the fork. Assembly of the pre-IC and origin melting are accompanied by activation of the CMG helicase and polymerase recruitment. This requires that the MCM2–7 complex transitions from encircling dsDNA as part of the pre-RC to ssDNA as part of the replication fork. The CMG helicase translocates along the leading strand, supporting a model in which the DNA is unwound by steric exclusion from the Mcm2–7 central channel (Fig. 1; Fu et al. 2011). Replication of the leading and lagging strands is coordinated with helicase unwinding in a large protein complex called the replisome; together, the replicating DNA and replisome constitute the replication fork (Fig. 1; Johnson and O'Donnell 2005). Fork progression also must be coordinated with disassembly of nucleosomes ahead of the fork and re-establishment of nucleosomes and the chromatin state on newly synthesized DNA. Nucleosome deposition is coordinated with fork elongation by interactions between histone chaperones and fork components (Alabert and Groth 2012). In addition, chromatin marks must be re-established on newly synthesized histones (Alabert and Groth 2012).

In the past 5 years, several methodologies have been developed for the capture of active replication forks and assessment of associated protein components (Kliszczak et al. 2011; Sirbu et al. 2011, 2013; Lopez-Contreras et al. 2013; Alabert et al. 2014). Isolation of proteins bound to nascent DNA (iPOND) and DNA-mediated chromatin pull-down (DM-ChP) use the thymidine analog EdU and click chemistry to pull down DNA fragments into which EdU is incorporated (Kliszczak et al. 2011; Sirbu et al. 2011). Short pulses of EdU followed by fixation allow selective pull-down of components at active replication forks, although newly synthesized DNA behind the replication fork also may be recovered (Sirbu et al. 2011, 2013; Lopez-Contreras et al. 2013). An EdU pulse followed by a thymidine chase results in pull-down of mature chromatin marks and the responsible remodelers (Sirbu et al. 2011; Lopez-Contreras et al. 2013). A similar approach, termed nascent chromatin capture (NCC), incorporates biotin-dUTP rather than EdU into replicating DNA (Alabert et al. 2014). Combining these techniques with mass spectrometry methods has allowed for proteomics analysis of active, stalled, and collapsed replication forks as well as chromatin maturation (Kliszczak et al. 2011; Lopez-Contreras et al. 2013; Sirbu et al. 2013; Alabert et al. 2014).

These approaches have produced insights into repair at the replication fork. iPOND analysis of forks in the presence of hydroxyurea (HU) detected known checkpoint proteins at stalled forks (Sirbu et al. 2011, 2013). When fork collapse is induced by long HU exposure or ATR knockdown, high levels of DSB repair components are pulled down (Sirbu et al. 2011, 2013). An interesting finding emerging from fork component characterization is that several checkpoint and repair proteins are detected at replication forks in the absence of damaging conditions (Sirbu et al. 2011, 2013; Alabert et al. 2014). These results raise the interesting possibility that forks are poised to deal with stalling throughout S phase. This property may be essential as forks proceed through difficult to replicate regions of the genome and for timely response to DNA damage and exogenous fork stress. However, it cannot be excluded that the repair proteins detected in these studies localize to spontaneously damaged forks in the population. This latter explanation is made less likely by the expectation that the proportional contribution of such forks would to be low.

## Impediments to replication fork progression

Once replication forks are established, there are numerous challenges that the forks may face before replication is completed. DNA damage such as interstrand cross-links cannot be unwound, and protein–DNA cross-links form barriers to the CMG helicase (Fu et al. 2011); these lesions require specialized pathways for repair and/or bypass (Duxin et al. 2014; Zhang et al. 2015). Other forms of damage, such as UV- and methyl methanesulfonate (MMS)-induced damage, block replication and cause uncoupling of the CMG helicase and polymerases (Byun et al. 2005), similar to chemical inhibition of polymerase activity by aphidicolin. Additionally, the dNTP and histone supply must be coordinated with fork elongation for proper S-phase progression and fork stability (Nelson et al. 2002; Mantiero et al. 2011; Poli et al. 2012; Mejlvang et al. 2014). It has been shown that disruption of the origin activation timing program leads to dNTP depletion, causing slowed fork elongation, fork stalling, and checkpoint activation (Mantiero et al. 2011; Poli et al. 2012). These studies highlight the importance of the replication program in coordinating fork elongation with a steady supply of raw materials for DNA synthesis.

It has been observed widely that specific regions of the genome are particularly prone to damage in the presence of replication stress, indicating that endogenous characteristics of the DNA sequence and/or chromatin structure can be problematic for fork progression; these regions are termed fragile sites. Fragile sites are defined formally as positions of constriction or breakage on metaphase chromosomes after exposure to replication stress (Ozeri-Galai et al. 2012) and can be subdivided into rare fragile sites (RFCs) and common fragile sites (CFSs). CFSs are positions that exhibit fragility across most individuals of a population, and the frequency of breakage is referred to as CFS expression (Debatisse et al. 2012). It is thought that CFSs are inherently difficult to replicate, as CFS expression is seen when the ATR checkpoint is inhibited in the absence of exogenous stress (Ozeri-Galai et al. 2012). Similarly, replication slow zones (RSZs) in yeast are prone to fork stalling and DNA breaks in the absence of the ATR homolog Mec1 (Ozeri-Galai et al. 2012). However, increased fork stalling is not observed across all metazoan CFSs (Debatisse et al. 2012; Ozeri-Galai et al. 2012). Instead, there is a collection of characteristics that are common but not universal among expressed CFSs: slow fork progression and/or frequent fork stalling, actively transcribed genes during replication, late replication timing, and lack of replication origins (Debatisse et al. 2012; Ozeri-Galai et al. 2012).

DNA sequence can impact CFS expression in several ways. Various forms of repetitive DNA can form DNA secondary structures in the ssDNA formed on the lagging strand during replication and block the replication fork (Mirkin and Mirkin 2007). Slow-replicating CFSs contain AT dinucleotide repeats (Ozeri-Galai et al. 2012), which exhibit hyperflexibility and can form secondary structures (Mirkin and Mirkin 2007). Replication forks frequently stall at these AT repeats and lead to DNA breaks in the absence of replication stress, and stalling is enhanced in the presence of aphidicolin (Ozeri-Galai et al. 2012). Secondary structures formed by trinucleotide repeats cause fork pausing and reversal and frequently correspond to break formation (Follonier et al. 2013; Liu et al. 2013; Gerhardt et al. 2014). One extensively studied example is G quadruplexes (G4s), highly stable secondary structures that form at stretches of G-rich DNA. G4s have been implicated in regulating gene expression (Maizels and Gray 2013) and origin selection (Besnard et al. 2012; Hoshina et al. 2013; Valton et al. 2014) in metazoan cells yet paradoxically pose a threat to genome stability by blocking replication forks. Replication across G4s requires specialized helicases such as FANCJ (London et al. 2008; Wu et al. 2008; Schwab et al. 2013) and Pif1 (Sanders 2010; Paeschke et al. 2011).

Fragile sites are prevalent in *Drosophila* endocycling tissues. The endocycle is a cell cycle variant composed of consecutive S and G phases in the absence of mitosis, resulting in increased cell ploidy (Smith and Orr-Weaver 1991). Replication of heterochromatin is actively repressed during the endocycle. This reduces the copy number of heterochromatic sequences compared with the overall cell ploidy and is known as underreplication. Certain euchromatic regions also are underreplicated during the endocycle in a tissue-specific manner (Nordman et al. 2011; Sher et al. 2012; Yarosh and Spradling 2014). In *Drosophila*, DNA damage and generation of DSBs lead to phosphorylation of the histone variant H2Av (Madigan et al. 2002), similar to H2AX in mammals (Rogakou et al. 1998); the phosphorylated histone is referred to as γH2Av. γH2Av is present throughout underreplicated sites of salivary gland chromosomes, showing that there is persistent DSB formation at these sites (Andreyeva et al. 2008; Nordman et al. 2014). Additionally, the observation that γH2Av is present across entire underreplicated domains rather than at the borders indicates that replication forks are not completely blocked but destabilized as they progress through these regions (Nordman et al. 2014).

Underreplication is dependent on the intriguing suppressor of underreplication (SUUR) protein, and *SuUR* mutants both restore copy number and alleviate DNA damage (Belyaeva et al. 1998; Andreyeva et al. 2008; Nordman et al. 2011, 2014; Sher et al. 2012). The SUUR protein has been demonstrated to be a dosage-sensitive inhibitor of fork progression that tracks with replication forks in particular genomic regions (Nordman et al. 2014). It appears that SUUR acts by destabilizing replication forks, but the underlying mechanism as well as the control of positional specificity await elucidation. Although full-length SUUR has no known human homologs, the N terminus is homologous to the SWI/SNF family ATPase/helicase domain (Makunin et al. 2002). However, residues essential for ATP binding and hydrolysis are not conserved, and thus SUUR may act as a decoy. It is an intriguing possibility that catalytically dead SWI/SNF homologs could function in other organisms to regulate fork progression during development.

## Detection of fork stalling and repair of collapsed replication forks

Obstructions to replication fork progression cause fork stalling and increase the likelihood of fork collapse and breakage. Although stalled forks can resume replication once the barrier or fork stress is alleviated, replication fork stalling can lead to uncoupling of the CMG helicase and DNA polymerases (Byun et al. 2005). Uncoupling results in extended RPA binding to exposed ssDNA that initiates a checkpoint response (Zou and Elledge 2003; Byun et al. 2005). ATR binds to the RPA-coated ssDNA via its binding partner, ATRIP (Zou and Elledge 2003). TopBP1 (Mus101 in *Drosophila* and Dbp11 in yeast) signaling from stalled forks recruits the Rad9–Rad1–Hus1 (9-1-1) complex (Yan and Michael 2009), and interaction with Rad9 facilitates activation of ATR by TopBP1 (Kumagai et al. 2006; Delacroix et al. 2007; Lee et al. 2007). iPOND and NCC experiments raised the possibility that TopBP1 travels with elongating forks in the absence of fork stress (Sirbu et al. 2013; Alabert et al. 2014), poising it as a first responder to replication stress. ATR activation leads to phosphorylation of several substrates in the DNA damage response, including Chk1 (Liu et al. 2000). Activated Chk1 then prevents initiation of origins near stressed replication forks (Ge and Blow 2010). Additionally, the GINS subunit Psf1 is phosphorylated by ATR in response to HU; thus, the checkpoint may function to regulate replisome function rather than stability (De Piccoli et al. 2012).

If stalled forks cannot be restarted, the replication machinery can disassemble. Several events can ensue at the fork. Electron microscopy (EM) studies in yeast found that HU treatment in the absence of Rad53/Chk2 leads to the accumulation of ssDNA at replication forks and reversed forks (Sogo et al. 2002). These reversed forks are known as “chicken foot” structures. Fork reversal was long thought to be the result of failed checkpoint response to fork stalling, but a recent study in human cell culture demonstrated that fork reversal is a common response to various replication perturbations when the checkpoint is intact (Zellweger et al. 2015). Fork reversal also is observed at trinucleotide repeats (Follonier et al. 2013), suggesting that chicken foot structures can form during unperturbed replication at hard to replicate sequences. Formation of reversed forks is dependent on PARP-1 regulation of the RECQ1 helicase as well as Rad51 (Zellweger et al. 2015). Other studies additionally have demonstrated a role for Rad51 and other homologous recombination (HR) components in fork stabilization independent of DSB repair (Lomonosov et al. 2003; Petermann et al. 2010; Schlacher et al. 2011; Hashimoto et al. 2012).

Cleavage of stalled or regressed forks can cause the fork to collapse and generate single-ended DSBs (see Fig. 3B) that require the DSB repair response. Several components are recruited to the break site upon DSB formation. Mre11–Rad50–Nbs1 (MRN; MRX in yeast) binds to DSBs and recruits ATM (Lee and Paull 2005). Inactive ATM forms a dimer; recruitment to DSBs leads to autophosphorylation and dimer dissociation, activating the kinase activity of the two monomers (Bakkenist and Kastan 2003). Upon activation, ATM phosphorylates multiple DSB response targets, including Chk2 and the histone variant H2AX (Rogakou et al. 1998; Ahn et al. 2000; Matsuoka et al. 2000). H2AX is phosphorylated in response to DSB formation for up to several megabases on either side of the break in mammalian cells (Rogakou et al. 1998; Madigan et al. 2002; Iacovoni et al. 2010), and γH2AX serves as a docking platform for DSB repair proteins (Celeste et al. 2002, 2003; Ward et al. 2003). Although *Saccharomyces cerevisiae* lacks an H2AX variant, either of the two H2A histones can be phosphorylated on Ser129 in response to DNA damage for up to 50 kb on either side of a DSB (Redon et al. 2003).

**Figure 2.**
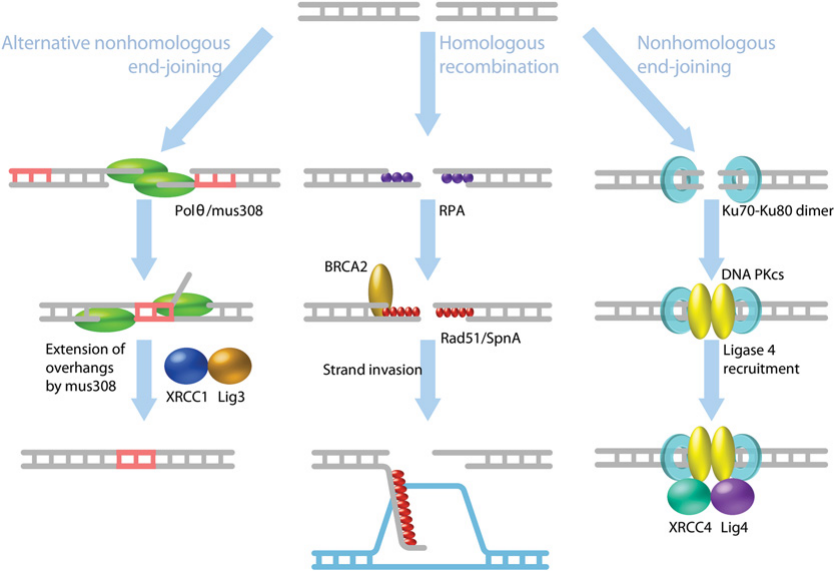
Pathways of DSB repair. Resection of the DSB commits repair to HR or alternative end-joining (alt-EJ) repair pathways. (*Left*) Alt-EJ by microhomology-mediated end-joining (MMEJ) requires Pol θ to align or template short microhomologies, generating deletions and insertions. The ends are then ligated together by the Ligase III (Lig3)/XRCC1 complex. (*Middle*) HR repair requires BRCA2 to recruit Rad51 and facilitates filament formation along the resected DNA. Rad51 filaments search for homologous sequences and initiate strand invasion to restore the exact sequence to the break site. (*Right*) Resection is blocked in nonhomologous end-joining (NHEJ) by Ku70–80 binding. The Ku70–80 heterodimer recruits the DNA-dependent protein kinase catalytic subunit (DNA-PKcs), and this complex brings the broken DNA ends together. The XRCC4–DNA Ligase IV (Lig4) complex is recruited and catalyzes DSB ligation. (Illustration by Steven Lee, http://www.graphiko.com.)

We first discuss the multiple pathways to repair DSBs in which two ends on either side of the DSB participate in the repair event. Pathway decision is ultimately determined by resection of the ends resulting from the DSB, with long 3′ overhangs facilitating HR (Fig. 2). Both the phase of the cell cycle and levels of accessory proteins influence whether nucleases have access to the DSB, thus dictating which repair pathway predominates. During S phase, when replication forks are actively elongating daughter DNA strands, S-phase CDK activity promotes break resection (Aylon et al. 2004; Ferreira and Cooper 2004; Ira et al. 2004; Bennardo et al. 2008; Yun and Hiom 2009; Chen et al. 2011; Tomimatsu et al. 2014). CDK promotes activity of the exonuclease CtIP, which, with MRN, mediates limited resection of the DSB ends to expose 3′ ssDNA overhangs (Bennardo et al. 2008; Yun and Hiom 2009). Extensive break resection also is mediated by CDK activity via EXO1 (Chen et al. 2011; Tomimatsu et al. 2014). In addition to CDK regulation, resection is dictated by competition between BRCA1 and 53BP1 for access to the DSB. Both BRCA1 and 53BP1 are concentrated at DSBs by γH2AX (Celeste et al. 2003; Ward et al. 2003) but also antagonize each other's recruitment (Escribano-Diaz et al. 2013). BRCA1 forms a complex with CtIP during S and G2 and promotes DSB resection (Yu and Chen 2004; Yun and Hiom 2009; Escribano-Diaz et al. 2013). During G1 of the cell cycle, 53BP1 prevents BRCA1 focus formation and thereby may inhibit CtIP access to DSBs to thus block resection-mediated repair (Escribano-Diaz et al. 2013). It therefore seems likely that the antagonistic relationship between 53BP1 and BRCA1 helps to integrate cell cycle regulation of DSB repair pathway choice.

Limited resection by MRN and CtIP commits the break to HR or alternative end-joining (alt-EJ) repair pathways (Yun and Hiom 2009; Truong et al. 2013). More extensive resection by EXO is required for HR repair (Yun and Hiom 2009; Truong et al. 2013). During HR repair, ssDNA exposed by resection is coated by Rad51, which mediates the search for homologous sequences and strand invasion for template repair (Fig. 2; McIlwraith et al. 2000). If only limited resection of the break occurs, alt-EJ can be used to repair the break (Yun and Hiom 2009; Truong et al. 2013). One form of alt-EJ is microhomology-mediated end-joining (MMEJ), which joins together microhomologies exposed by resection. MMEJ requires Pol θ, which binds to ssDNA on both ends of a DSB and aligns short 4- to 10-base-pair microhomology sequences (Fig. 2; Chan et al. 2010; Kent et al. 2015; Mateos-Gomez et al. 2015). Microhomologies also can be generated by Pol θ, resulting in insertions templated from sequences outside the break site (Chan et al. 2010; Yu and McVey 2010; Hogg et al. 2012; Kent et al. 2015). MMEJ is a highly error-prone pathway, generating deletions and insertions at the break site from microhomology alignment and extension or complex chromosome rearrangements (Chan et al. 2010; Yu and McVey 2010; Hogg et al. 2012; Kent et al. 2015; Mateos-Gomez et al. 2015; Sakofsky et al. 2015).

The nonhomologous end-joining (NHEJ) repair pathway directly joins the two broken ends of a DSB and actively prevents 3′ resection (Fig. 2). The blunt DNA ends are bound by the Ku70–80 heterodimer and DNA-dependent protein kinase catalytic subunit (DNA-PKcs), which prevent resection and promote association of the broken ends (Dvir et al. 1992; Gottlieb and Jackson 1993; Yoo and Dynan 1999; Pierce et al. 2001; Walker et al. 2001; Graham et al. 2016). Ligase IV (Lig4) catalyzes ligation of the DSB ends, and this reaction is enhanced by XRCC4 (Grawunder et al. 1997). Direct ligation of the broken ends often generates small deletions at the break site (Jeggo 1998), and thus NHEJ is considered an error-prone repair mechanism. NHEJ is active throughout the cell cycle, but competition from resection-mediated pathways during S and G2 make it more prevalent during G1.

Break-induced replication (BIR) is a subtype of HR repair that can account for repair of a DSB with only one end. It was characterized in yeast following the observation that one end of a broken chromosome can copy a homologous template to the end of the chromosome. BIR requires many of the components present at S-phase replication forks but does not require the pre-RC components ORC or Cdc6 (Lydeard et al. 2010). These results support the hypothesis that BIR establishes processive replication forks in the absence of an origin. In addition to canonical fork requirements, the appearance of BIR repair products depends on the nonessential Pol δ subunit Pol32 (Lydeard et al. 2007), and the Pif1 helicase is required for long-range synthesis during BIR (Saini et al. 2013; Wilson et al. 2013; Vasianovich et al. 2014).

BIR also was demonstrated in human cell lines under conditions of replication stress, suggesting that it is used to repair collapsed replication forks (Costantino et al. 2014). The investigators found that BIR generated duplications and rearrangements. A model for the generation of copy number variations proposed a form of BIR that relies on microhomology annealing, termed microhomology-mediated BIR (MMBIR), in repair of collapsed replication forks (Hastings et al. 2009). Complex rearrangements and copy number variations consistent with BIR and MMBIR are observed across human cancers and other genomic diseases (Hastings et al. 2009).

## Rereplication: how origin deregulation impairs fork integrity

Origin refiring in a single S phase activates the DNA damage checkpoint, generates DSBs, and causes DNA fragmentation (Mihaylov et al. 2002; Melixetian et al. 2004; Zhu et al. 2004; Archambault et al. 2005; Green and Li 2005; Davidson et al. 2006; Zhu and Dutta 2006; Finn and Li 2013; Neelsen et al. 2013), making rereplication a highly genotoxic event. The damage caused by origin refiring appears to arise from problems at the rereplication forks. Rereplication forks exhibit inhibited elongation and progress only 30–35 kb from the origin in yeast (Nguyen et al. 2001). Consistent with this observation, rereplication does not result in full replication of the genome and generates cells with ploidies between integral doubling values (Melixetian et al. 2004; Zhu et al. 2004; Green and Li 2005). If the DNA damage checkpoint is blocked, cells enter mitosis with partially rereplicated DNA, resulting in cells with sub-G1 ploidy (Mihaylov et al. 2002) and chromosome breaks and fusions (Melixetian et al. 2004).

To prevent this catastrophic damage, replication initiation is tightly regulated with the cell cycle to ensure that each origin fires only once per cell cycle (Tanaka and Araki 2013). In budding yeast, CDK activity prevents rereplication by inhibiting multiple components of the pre-RC at several levels of regulation. Phosphorylation of Orc2 and Orc6 by CDK prevents pre-RC formation (Arias and Walter 2007). CDK phosphorylation events inhibit *cdc6* transcription. Direct phosphorylation of Cdc6 promotes ubiquitination by SCF to lead to its degradation by the proteasome from late G1 to S phase and then, in mitosis, prevents Cdc6 from loading Mcm2–7 (Arias and Walter 2007). Finally, CDK phosphorylation exports Cdt1 and Mcm2–7 from the nucleus (Arias and Walter 2007).

CDK activity also prevents rereplication in metazoans by targeting multiple pre-RC components, although the mechanisms differ between organisms. One common and major regulator of pre-RC activity is geminin, which binds to and sequesters Cdt1 to prevent Mcm2–7 from being loaded at origins (Arias and Walter 2007). Depletion of geminin is sufficient to induce rereplication in *Drosophila* and human cultured cells (Mihaylov et al. 2002; Melixetian et al. 2004; Zhu et al. 2004; Zhu and Dutta 2006). Overexpression of its target, Cdt1, in human cells and *Drosophila* and addition of recombinant Cdt1 to *Xenopus* cell extracts also cause rereplication (Arias and Walter 2007).

**Figure 3.**
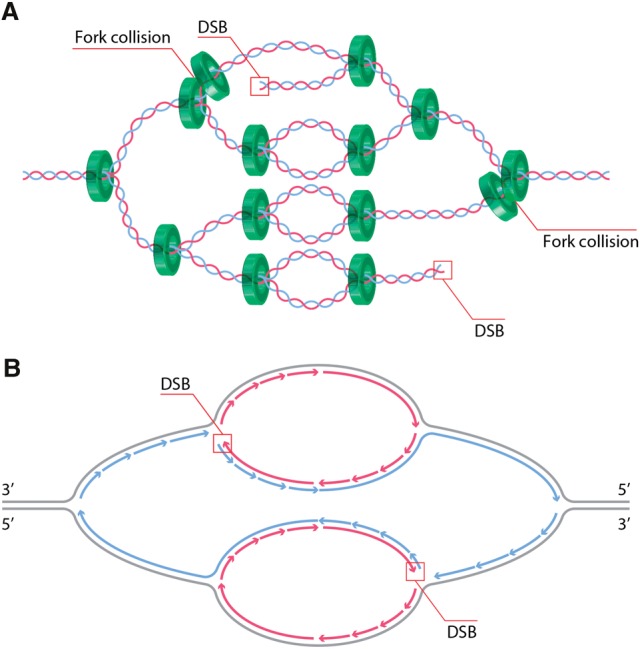
The fork collision model of DSB generation by rereplication. (*A*) Collisions between two replication forks on the same DNA duplex (green) would generate a DSB behind the second replication fork (arrows). Multiple origin reinitiations would increase the frequency of fork collisions and thus the number DSBs formed. Fork collisions are expected to be stochastic and may occur at only a subset of forks, as shown here. (*B*) One proposed mechanism of DSB formation. If the leading strand (red) from a second fork collides with unligated Okazaki fragments on the lagging strand of an earlier fork (blue), a DSB results. Note that, although this is single-end DSB, fork collisions on opposite sides of the origin could generate two ends that could be joined by NHEJ. Other mechanisms such as exonuclease cleavage or steric breakage of the DNA also could generate additional DSB ends. (Illustration by Steven Lee, http://www.graphiko.com.)

DSBs and chromosome fragmentation generated during rereplication are consistent with predicted products of head-to-tail collisions between adjacent replication forks (Fig. 3A; Davidson et al. 2006). If a leading strand reaches a region with unligated Okazaki fragments on a fork in front of it, this results in a DSB (Fig. 3B). This is supported by the observation that broken DNA fragments are generated around an origin after rereplication is induced (Finn and Li 2013). The pattern of repeat expansion during rereplication also is consistent with a “forks chasing forks” model of DSB formation (Green et al. 2010; Finn and Li 2013). Such collisions require that rereplication forks can progress faster along the newly synthesized DNA and thus catch up with the forks in front of them (Davidson et al. 2006). Indeed, nascent DNA is in an immature chromatin state for up to 20 min after replication and is more susceptible to nuclease degradation (Hildebrand and Walters 1976). Immature chromatin behind the first replication fork could thus be easier to disassemble and more susceptible to breaks as the rereplication forks arrive.

Strong evidence for the fork collision model (Fig. 3) was provided by recent studies in *Drosophila* follicle cells. The follicle cells of *Drosophila melanogaster* undergo rereplication from defined origins as a developmental strategy to enhance protein production by gene amplification. Rereplication occurs at six loci, termed *Drosophila amplicons in follicle cells* (*DAFC*s) (Kim et al. 2011). The precise timing and location of origin firing at the *DAFC*s enables isolation of replication forks at specific points after origin initiation and real-time tracking of fork progression (Claycomb and Orr-Weaver 2005). Additionally, replication forks can be visualized directly by introducing a nucleotide analog (Claycomb and Orr-Weaver 2005), providing the necessary resolution to observe events occurring at sites of active replication. Analysis of rereplication damage at the *DAFC*s revealed that the DSB marker γH2Av tracks with EdU at the *DAFC*s and is dependent on ATR and ATM activity (Alexander et al. 2015). These data are supported by ChIP-seq (chromatin immunoprecipitation [ChIP] combined with high-throughput sequencing) measurements showing that γH2Av is enriched over positions of active rereplication forks in the follicle cells (Alexander et al. 2015). The *DAFC* system thus has made it possible to detect damage present at known positions of rereplication at defined points in time after the onset of origin refiring with a resolution not possible in previously studied systems. Additionally, the data indicate that DNA damage and DSBs are formed at the active replication forks, where head-to-tail collisions could occur.

Other data suggest that DSBs formed by rereplication occur in the absence of fork collisions. RNAi depletion of *Emi1* (an APC/C inhibitor that prevents geminin degradation during S and G2) (Machida and Dutta 2007) in human cells in culture generates ssDNA gaps along the DNA before detectable rereplication occurs (Neelsen et al. 2013). The investigators proposed that deregulated origin firing leads to unrepaired gaps in the first round of replication, which cause fork collapse and DNA fragmentation when rereplication forks meet these gaps on the template strand. Gaps also were reported when recombinant Cdt1 was added to *Xenopus* extracts, but the appearance of gaps in relation to the onset of rereplication was not reported (Neelsen et al. 2013). It is therefore possible that the cause of DSBs during rereplication is dependent on the mechanism and timing of origin deregulation.

The generation of DSBs during rereplication poses the question of how these breaks are repaired. Although fork collision results in a DSB with a single end, DSBs with two ends can be generated if fork collision occurs on both sides of the origin (Fig. 3B) and by several other mechanisms detailed by Truong et al. (2014). Thus, the pathways for repair of DSBs with two ends (Fig. 2) as well as BIR potentially can be involved in repair. In human cells in culture, Rad51 foci form after geminin depletion (Melixetian et al. 2004; Zhu and Dutta 2006), suggesting that the HR repair pathway is activated to repair broken forks. Another study using human cells reported that 53BP1 foci appear overlapping with γH2AX when rereplication is induced, suggesting NHEJ repair (Neelsen et al. 2013). However, these studies only reported on markers of one repair pathway and did not test whether there is a preferred mechanism of repair. In two human cell culture lines, knockdown of the HR components Rad51, BRCA1, and CtIP reduced cell proliferation when Cdt1 was overexpressed, whereas knockdown of the NHEJ components Ku70 and XRCC4 had no effect (Truong et al. 2014). Interestingly, knockdown of the MMEJ component Lig3 also reduced proliferation, although to a lesser extent than HR factors (Truong et al. 2014). Using GFP reporter constructs for HR and MMEJ repair after rereplication, the investigators found that the percentage of GFP-positive cells increases upon Cdt1 overexpression; this frequency is not altered by knockdown of Ku70 or XRCC4, suggesting that NHEJ does not compete for repair in these cells (Truong et al. 2014).

Evidence from studies in *S. cerevisiae* also suggests that multiple pathways can repair rereplication forks. The HR pathway mutants *rad52* and *rad59* are synthetically lethal in rereplicating strains, as are the three components of the MRX complex (*mre11*, *rad50*, and *xrs1*) (Archambault et al. 2005). In budding yeast, the MRX complex is involved in both NHEJ and HR repair (D'Amours and Jackson 2002). Notably, mutants with lesions in the NHEJ components *yku70*, *yku80*, and *dnl4* (*lig4*) are viable in rereplicating strains, suggesting that MRX is functioning in HR repair during rereplication (Archambault et al. 2005). However, a recent study showed that the frequency of rereplication-induced aneuploidy is halved in *rad52* mutants and tripled in *dnl4* mutants; these results demonstrate that both the HR and NHEJ pathways are active and compete to repair DSBs generated by rereplication (Hanlon and Li 2015). Finally, repeat expansion after rereplication in *S. cerevisiae* occurs via single-strand annealing (SSA) repair and is genetically dependent on *rad52*, *rad1*, and *msh3* but not *rad51* or *dnl4* (Green et al. 2010; Finn and Li 2013).

Use of multiple pathways in rereplication-induced DSB repair also is supported by studies in *Drosophila* follicle cells. Multiple rereplication events at the *DAFC*s generate gradients of amplification, which are easily visualized by array comparative genomic hybridization (aCGH) analysis (Kim et al. 2011). The shape of the amplification gradients generated by aCGH is reflective of replication fork progression, in which rapid decreases in copy number along the gradients indicate that fork progression is impaired. aCGH thus can be used to measure fork movement in the absence of various DSB repair components. Because unrepaired DSBs within the *DAFC*s block all subsequent replication forks on the same strand from moving beyond the break site, removal of DSB repair pathways used during rereplication reduces overall fork progression. Changes in global fork progression were quantified by half-maximum distance analysis, which measures the distance between the left and right sides of half the maximum copy number of the aCGH gradients (Alexander et al. 2015). This approach was used to analyze mutants in DSB repair pathways. NHEJ is required for repair of these DSBs, as the absence of the NHEJ factor Lig4 inhibits fork progression across the *DAFC*s (Alexander et al. 2015). Additionally, the MMEJ component Pol θ is required for complete fork progression at only some of the *DAFC*s, revealing that the use of this pathway for rereplication DSB repair is site-specific (Alexander et al. 2016). In contrast, the absence of the HR components Brca2 and Rad51 enhances fork movement (Alexander et al. 2016). These results suggest that HR is active but cannot productively contribute to DSB repair before the end of amplification, which occurs over a 7.5-h time in development. As a consequence, HR appears to compete with and impede NHEJ repair. There is a possible contribution of BIR at the *DAFC*s because replication fork progression is reduced in *pol32* and *pif1* mutants (Alexander et al. 2016), but because these two proteins participate in other aspects of fork progression, it cannot be excluded that these functions account for the mutant phenotype (Alexander et al. 2016). Together, the analysis of *DAFC*s suggests that DSB repair pathway choice following rereplication is governed by genomic position, reaction kinetics, and repair pathway competition.

## Conclusions and outlook

Recent studies in yeast and *Drosophila* have permitted the analysis of rereplication at specific genomic sites and thus demonstrated directly that rereplication can lead to fork collision and formation of DSBs. Although there are parallels between these fork collision events and fork stalling during normal DNA replication, such as the involvement of DNA damage signaling pathways, the extent to which repair mechanisms are shared remains to be determined. For example, it is unknown whether fork reversal occurs at rereplication fork collision sites and whether it affects fork stability. The analyses of rereplication suggest that a variety of pathways can be used to repair DSBs resulting from fork collision. Resection-dependent pathways, including HR, SSA, and MMEJ, are the most commonly observed, consistent with rereplication occurring in S and G2 phases of the cell cycle, when resection is most efficient. However, NHEJ repair also has been reported (Neelsen et al. 2013; Alexander et al. 2015; Hanlon and Li 2015). Therefore, as with general DSBs, pathway choice for repair of rereplication DSBs could be the result of pathway competition influenced by the cell cycle phase and exonuclease access to the break site.

Mounting evidence suggests that repair choice influences the consequences for genome instability. Refiring within repetitive sequences is repaired by SSA and leads to copy number expansions (Green et al. 2010; Finn and Li 2013). Rereplication near centromeres increases the rate of aneuploidy if forks are repaired by HR, whereas NHEJ seems to protect these cells from missegregation (Hanlon and Li 2015). These types of chromosomal aberrations are common across numerous human cancers (Abbas et al. 2013). Additionally, Cdt1 overexpression drives oncogenic transformation in cell culture and tumor formation in mouse models and is observed in various human cancer cell lines (Arentson et al. 2002; Karakaidos et al. 2004; Xouri et al. 2004; Seo et al. 2005). Together, these observations strongly suggest that the same mechanisms used to artificially induce rereplication in the laboratory and the resulting genome instability are physiologically relevant to cancer progression. However, rereplication has not been observed directly at specific sites in cancer cells.

Technological advances such as iPOND and rapidly improving deep sequencing platforms could help to shed light on which repair mechanisms are activated during rereplication and the resulting repair products. Proteomics would reveal which repair proteins are recruited to rereplication forks, providing a list of competing repair mechanisms. The continued study of model systems such as *S. cerevisiae* and the *Drosophila DAFC*s will make it possible to dissect both the mechanisms of rereplication fork repair and the consequences of pathway choice on genome stability.
